# Does set configuration influence long-term adaptations to complex training in national-level wrestlers? A volume-matched randomized trial

**DOI:** 10.7717/peerj.21438

**Published:** 2026-06-25

**Authors:** Yueqiang Ma, Pengpeng Gou, Yuxiong Huang, Jialiang Yu, Lijia Zhou, Kai Xu, Binbin Jia, Danyang Li

**Affiliations:** 1 School of Sports Training, Wuhan Sports University, Wuhan, China; 2 Center of Strength and Conditioning, Wuhan Sports University, Wuhan, China; 3 Department of Sports and Health Sciences, Academy of Wellness and Human Development, Faculty of Arts and Social Sciences, Hong Kong Baptist University, Hong Kong, China; 4 School of Athletic Performance, Shanghai University of Sport, Shanghai, China; 5 School of Medical and Health Sciences, Edith Cowan University, Perth, Australia

**Keywords:** Complex training, Cluster set, Explosive power, Wrestling, Strength development

## Abstract

**Objectives:**

Complex training is commonly used to enhance strength and explosive performance in athletes; however, the long-term influence of set configuration within complex training remains unclear in highly trained wrestlers. This randomized, volume-matched trial investigated whether set configuration influences long-term adaptations to complex training (CT) in national-level male Chinese-style wrestlers. Specifically, the study compared the effects of cluster-set complex training (CS-CT) and traditional-set complex training (TS-CT) on maximal strength, lower-limb explosive performance, anaerobic power, and sport-specific performance.

**Methods:**

Sixteen highly trained wrestlers (Tier 3; *n* = 16) completed an 8-week lower-body CT program performed twice weekly. Participants were randomly allocated to either CS-CT (*n* = 9) or TS-CT (*n* = 7). Training volume, intensity progression (70–85% 1RM), and exercise selection were identical between groups, differing only in set configuration. Primary outcomes included back squat one-repetition maximum (1RM), countermovement jump (CMJ), squat jump (SJ), drop jump (DJ), standing long jump (SLJ), Wingate anaerobic performance, and wrestling-specific field tests. Group × time interaction effects were analyzed using Generalized Estimating Equations.

**Results:**

Both CS-CT and TS-CT produced significant improvements in back squat 1RM (both *p* < 0.05). The CS-CT group demonstrated significant within-group improvements in SLJ, SJ, DJ, and selected sport-specific tests (*p* < 0.05), whereas the TS-CT group showed improvements in CMJ and hip-rotation kick performance (*p* < 0.05). However, no significant group × time interaction effects were observed for any performance variable (all *p* > 0.05).

**Conclusions:**

When total training volume and intensity are matched, complex training effectively enhances strength and explosive performance in national-level wrestlers. However, modifying set configuration (cluster *vs.* traditional) did not result in clearly superior long-term adaptations over an 8-week training period in highly trained athletes.

## Introduction

Wrestling is a highly demanding combat sport that places substantial requirements on athletes’ physical capacities, including high levels of muscular strength, speed, endurance, coordination, and, in particular, explosive power (Chaabene et al., 2017). Explosive power is essential for the successful execution of decisive technical actions such as throws, chest-to-chest techniques, lifting maneuvers, and defensive responses (Bai et al., 2024). Accordingly, training strategies aimed at enhancing explosive power are considered fundamental for improving competitive performance in wrestlers (Horswill, 1992). Traditional resistance training constitutes the cornerstone for the development of muscular strength, whereas plyometric training has been widely recognized as an effective means of improving explosive performance (Marshall et al., 2021). In recent years, complex training (CT), an advanced training strategy that integrates these two modalities, has attracted increasing attention within the field of sport science (Cormier et al., 2022). In contrast to conventional resistance training programs that primarily emphasize a single dimension of strength development, complex training typically involves the execution of a heavy-load resistance exercise followed immediately by a biomechanically similar, low-load plyometric or explosive movement within the same training session (Cormier et al., 2020; Docherty & Hodgson, 2007). Through this sequencing, complex training simultaneously targets both strength and explosive power (Cormier et al., 2020; Docherty & Hodgson, 2007; Marshall et al., 2021). The principal advantage of this approach lies in its enhanced training efficiency, and accumulating evidence indicates that complex training can elicit positive adaptations in vertical jump performance, sprint ability, and strength development (Aksović et al., 2025; Cormier et al., 2020).

The theoretical foundation of complex training is largely attributed to the phenomena of post-activation potentiation (PAP) and, more recently, post-activation performance enhancement (PAPE) (Gautam, Singh & Varghese, 2024; Hodgson, Docherty & Robbins, 2005). The underlying mechanism is based on the premise that an initial high-load conditioning activity acutely enhances neuromuscular excitability, thereby potentially improving the performance of subsequent explosive movements (Docherty & Hodgson, 2007; Gautam, Singh & Varghese, 2024; Marshall et al., 2021). However, the potentiation effect associated with classical PAP is relatively short-lived, with a reported half-life of approximately 28 s (Blazevich & Babault, 2019; Cormier et al., 2022). In contrast, improvements in power output or movement performance observed during complex training practice often emerge over a longer time window, such as 6–10 min following the conditioning activity (Cormier et al., 2022). Accordingly, PAPE may be more relevant than classical PAP when interpreting complex training in applied training settings. Despite the evident advantages of complex training, its traditional implementation is not without limitations. A critical concern is that the heavy-load conditioning activity used to induce PAPE may itself generate substantial neuromuscular fatigue (Docherty & Hodgson, 2007; Gautam, Singh & Varghese, 2024; Marshall et al., 2021). If this fatigue is not effectively managed, it may offset or even mask the potential potentiation effects, thereby constraining the overall effectiveness of complex training, particularly during protocols involving multiple sets (Bakhshinejad et al., 2025; Garbisu-Hualde & Santos-Concejero, 2021; Huygaerts et al., 2020). Accumulated fatigue may not only reduce the quality and power output of subsequent explosive exercises but also increase the risk of musculoskeletal injury (Xenofondos et al., 2018; Yang, Zhang & Cai, 2025). Consequently, achieving an optimal balance between eliciting sufficient potentiation and minimizing fatigue accumulation represents a central challenge in the optimization of complex training.

Cluster-set training (CS), as an emerging resistance training strategy, has been proposed as a potential solution to this challenge (Tufano, Brown & Haff, 2017). Cluster sets involve the systematic insertion of brief intra-set rest intervals between individual repetitions or small clusters of repetitions within a set (Jukic et al., 2022; Tufano, Brown & Haff, 2017). Compared with traditional set configurations (traditional sets, TS), in which all repetitions are performed consecutively, cluster-set structures may attenuate fatigue accumulation by allowing partial phosphocreatine resynthesis and maintaining neuromuscular excitability during the training bout (Jukic et al., 2020). As a result, cluster-set training typically enables athletes to sustain higher movement velocity and power output throughout a set, particularly under moderate to heavy loading conditions (Latella et al., 2019; Tufano et al., 2016). In addition to its fatigue-mitigating effects, cluster-set training has been shown to acutely reduce cardiovascular strain while preserving velocity and power during resistance exercise (Tufano et al., 2016). Evidence further suggests that intra-set rest intervals as short as 6 s may be sufficient to induce partial recovery and enhance muscular power output (Jusoh, Ishak & Tufano, 2019). Given that complex training relies on high-quality, high-intensity conditioning activities to induce potentiation effects while inevitably generating fatigue, integrating cluster-set principles into complex training frameworks may yield synergistic benefits (Cuevas-Aburto et al., 2022). Specifically, employing cluster-set configurations during the heavy-load resistance component of complex training may reduce fatigue induced by high loads, thereby increasing the net potentiation effect and improving the quality and effectiveness of subsequent explosive exercises (Marshall et al., 2021; Nickerson et al., 2018). Moreover, applying cluster-set structures to the explosive exercise component may help maintain explosive performance across repeated efforts (Jukic et al., 2020).

To date, comprehensive literature searches have identified only two studies that have examined the combination of cluster-set configurations with complex training (Rong & Xiu, 2024; Treeraj, Kamutsri & Lawsirirat, 2016), and only one of these investigations implemented a long-term training intervention (Rong & Xiu, 2024). This limited body of evidence highlights a clear gap in the current literature. This gap is particularly relevant to wrestling, where athletes must repeatedly produce explosive actions under fatigue and within technically demanding movement patterns. Therefore, findings from other athletic populations may not directly reflect the training responses of highly trained wrestlers (Chaabene et al., 2017). Research specifically examining the effects of integrating cluster-set configurations into complex training on the development of explosive power in wrestlers remains scarce. Given that wrestlers must repeatedly express high levels of explosive power during competition, optimizing fatigue management and potentiation strategies is of critical importance for performance (Cieśliński, Gierczuk & Sadowski, 2021). Therefore, the primary purpose of the present study was to investigate the effects of an eight-week lower-body complex training program with different set configurations (cluster sets *vs.* traditional sets) on lower-body explosive performance in young, highly trained male Chinese-style wrestlers. It was hypothesized that, compared with traditional-set complex training (TS-CT), cluster-set complex training (CS-CT) would result in significantly greater improvements in measures of explosive performance.

## Methods

### Participants

All participants were from the Chinese national professional wrestling team. All athletes reported comparable weekly training loads prior to the commencement of the intervention. Eligibility criteria required participants to meet the following conditions: athletic caliber consistent with Tier 3 (Highly Trained/National Level) or higher according to the participant classification framework proposed by McKay et al. (2022), indicating long-term engagement in structured and periodized sport-specific training and participation in official competitions at the provincial or national level (McKay et al., 2022); a minimum of three years of systematic Chinese wrestling–specific training combined with resistance training experience; good overall health status with no history of serious lower-limb injury within the six months preceding the study. In addition, to ensure an adequate physical foundation for the intervention, all participants were required to demonstrate a back-squat one-repetition maximum (1RM) of at least 1.5 times their body mass. Athletes were excluded from the final analysis if, during the study period, they reported any acute or chronic musculoskeletal injury likely to compromise lower-limb force production or sport-specific technical performance. Participants were also excluded if they had a history of cardiovascular, respiratory, neurological, or metabolic disorders within the six months prior to study entry, or any medical condition contraindicating high-intensity training or maximal strength testing. Furthermore, the use of medications or supplements known to influence cardiovascular responses, neuromuscular function, or exercise tolerance (*e.g.*, β-adrenergic blockers, stimulants, glucocorticoids, or sedative–hypnotic agents) constituted grounds for exclusion. During the intervention, participants who missed two or more training sessions, failed to complete all prescribed training or testing procedures, or presented with incomplete key outcome data were not included in the final statistical analysis.

This study initially recruited 20 male Chinese wrestlers. During the intervention period, four participants were excluded from the final analysis due to incomplete training and testing data resulting from absences of two or more training sessions. Ultimately, 16 participants completed the study and were included in the final analysis (*n* = 16), with nine athletes in the CS-CT group and seven athletes in the TS-CT group.

### Experimental design

All participants provided written informed consent prior to participation in the study. This randomized controlled trial received ethical approval from the Survey and Behavioural Research Ethics Committee, The Wuhan Sport University (Protocol ID: 2025159) (Harriss, Jones & MacSween, 2022). Randomization was performed using a computer-generated allocation sequence prepared by an independent investigator, thereby ensuring appropriate allocation concealment (O’Donoghue, 2009).

**Figure 1:**
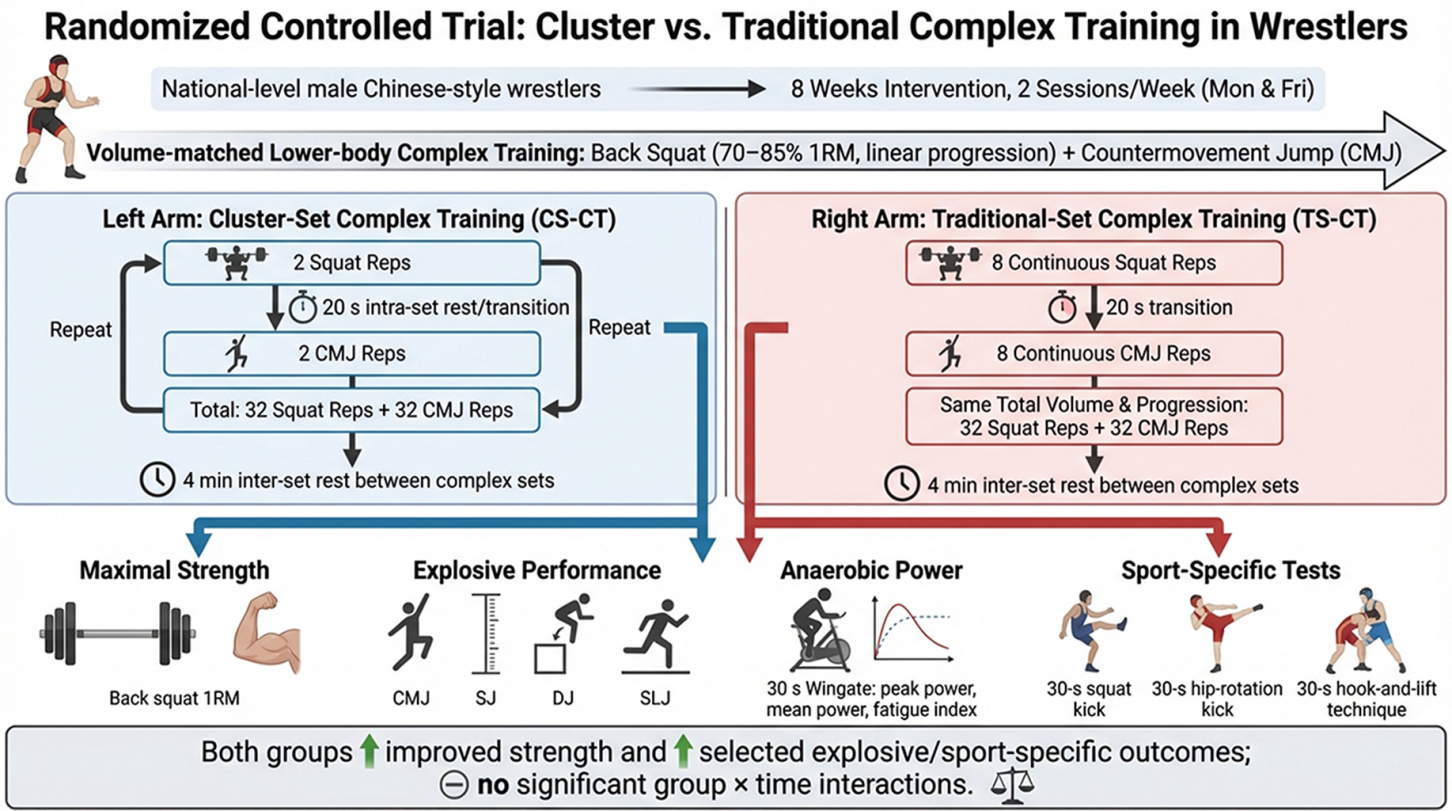
Experimental design of the volume-matched complex training intervention. National-level male Chinese-style wrestlers completed an eight-week lower-body complex training program performed twice weekly. Participants were randomly assigned to either cluster-set complex training (CS-CT) or traditional-set complex training (TS-CT). Both groups performed identical training exercises, volume, and intensity progression (70–85% one-repetition maximum (1RM)). The CS-CT protocol alternated two squat repetitions with two countermovement jumps (CMJ) separated by a 20 s intra-set interval. The TS-CT protocol consisted of eight continuous squat repetitions followed by eight CMJ repetitions after a 20 s transition interval. Both groups completed 32 squat repetitions and 32 CMJ repetitions per session with 4 min rest between complex sets. Outcome measures included maximal strength (back squat 1RM), explosive performance (CMJ, squat jump (SJ), drop jump (DJ), and standing long jump (SLJ)), anaerobic power (30-s Wingate test), and sport-specific wrestling performance tests.

**Figure 2:**
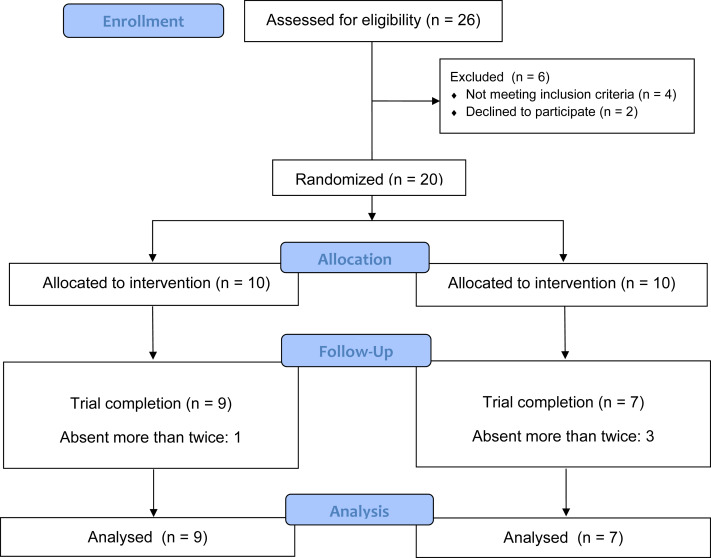
Flow diagram of participant recruitment, randomization, follow-up, and analysis. Participants were screened for eligibility (*n* = 26) and randomly allocated to cluster-set complex training (CS-CT) or traditional-set complex training (TS-CT). Six participants were excluded prior to randomization (four did not meet inclusion criteria and two declined to participate). Twenty athletes were randomized (10 per group). During the intervention, four participants were excluded from the final analysis due to absence from more than two training sessions. Ultimately, sixteen participants completed the study and were included in the statistical analyses (CS-CT: *n* = 9; TS-CT: *n* = 7).

A total of 20 participants were enrolled following online and on-site recruitment after meeting all predefined inclusion criteria. The intervention was conducted during the competitive phase of the athletes’ annual training cycle. To reduce potential measurement bias, all instrument-based assessments were administered by experienced evaluators who were blinded to group assignment. Participants were randomly allocated to either the CS-CT or TS-CT group and undertook an eight-week lower-body complex training (CT) intervention. Both groups followed identical training volume and progression schemes, with the sole distinction being the configuration of training sets. Outcome measurements were obtained before group allocation to establish baseline values and were reassessed upon completion of the eight-week intervention period. A schematic overview of the experimental design and study timeline is presented in Figs. 1 and 2.

### Measurement

#### Explosive performance

Lower-limb explosive performance was assessed using a battery of standardized jump tests, including the countermovement jump (CMJ), squat jump (SJ), drop jump (DJ), and standing long jump (SLJ). All jump tests were conducted following established testing protocols commonly adopted in athletic performance research. Participants completed three maximal trials for each test, with 30 s of passive recovery between trials, and the best performance was retained for analysis.

For CMJ, participants started from an upright standing position with hands placed on the hips, performed a rapid countermovement to a self-selected depth, and immediately executed a maximal vertical jump. SJ was performed from a static semi-squat position (∼90° knee flexion) without a preparatory countermovement to minimize the contribution of the stretch–shortening cycle. DJ was performed by stepping off a 40 cm platform onto a force plate, followed by an immediate maximal vertical rebound jump, with emphasis on minimizing ground contact time. Vertical jump height for CMJ, SJ, and DJ was recorded using a force platform system (Kistler Instrumente AG, Winterthur, Switzerland). SLJ was performed with a two-foot take-off and arm swing. Jump distance was measured from the take-off line to the nearest heel landing point. All tests were supervised by experienced assessors to ensure standardized execution.

#### Anaerobic power

Anaerobic power was evaluated using the 30 s Wingate Anaerobic Test conducted on a mechanically braked cycle ergometer (Monark Ergomedic 894E, Monark Exercise AB, Sweden). Seat height was individually adjusted to achieve approximately 25° knee flexion at the lowest pedal position.

Participants completed a standardized 5 min warm-up at 50 W, followed by three 5 s maximal sprints interspersed with 45 s of active recovery at 50 W to enhance neuromuscular readiness. After a 3 min passive rest, the 30 s Wingate test was initiated with a braking force equivalent to 0.075 kg kg^−^^1^ body mass. Participants were instructed to remain seated and pedal maximally throughout the test. Peak power (PP), mean power (AP), and fatigue index (power drop, PD) were calculated automatically by the ergometer software.

#### Sport-specific performance

Sport-specific performance was assessed using three wrestling-specific field tests: the 30 s squat kick test, 30 s hip-rotation kick test, and 30 s hook-and-lift technique test. All tests were performed on a flat indoor surface, and performance was quantified as the number of correctly executed repetitions completed within 30 s.

Participants were instructed on standardized movement patterns before testing, and each test was demonstrated by an experienced coach. Verbal encouragement was provided during testing to ensure maximal effort. These tests were selected to reflect lower-limb explosive capacity, coordination, and technical execution under time-constrained conditions.

#### Maximal strength

Lower-limb maximal strength was assessed using the 1RM back squat test. Participants completed a standardized warm-up consisting of 5 min of cycling, dynamic stretching, and progressive squat sets at approximately 50%, 70%, and 90% of self-reported 1RM. Load was subsequently increased until the maximal load that could be lifted once with proper technique was determined.

Rest intervals of 3–5 min were provided between attempts, with longer rest periods for heavier loads. All lifts were supervised by certified strength and conditioning specialists to ensure safety and technical validity.

### Intervention

Participants completed an eight-week lower-body complex training intervention performed twice per week on non-consecutive days (Monday and Friday). Both groups followed identical training frequency, exercise selection, total training volume, and intensity progression, differing only in set configuration. All training sessions were supervised by certified strength and conditioning specialists to ensure adherence to the prescribed protocol and correct execution of all exercises.

Each training session consisted of a complex training sequence pairing a heavy-load resistance exercise with a biomechanically similar explosive movement. The resistance exercise was the free-weight barbell back squat, and the explosive exercise was the CMJ. Participants were instructed to perform the concentric phase of all squat repetitions with maximal intended velocity to maximize neuromuscular activation and ensure consistency across sessions. Training intensity followed a linear progression model over the eight-week intervention period, increasing from 70% to 85% of individual 1RM. In each training session, participants completed a total of 32 squat repetitions and 32 CMJ repetitions, thereby ensuring equivalent training volume between groups.

In the cluster-set complex training group, the complex sequence consisted of two repetitions of the back squat followed by a 20 s intra-set rest interval and then two maximal CMJ repetitions. This sequence was repeated continuously until the prescribed number of repetitions was completed. No additional rest was provided between the squat and jump exercises beyond the predefined intra-set interval. A standardized inter-set rest period of 4 min was implemented between successive complex sets. In the traditional-set complex training group, participants performed eight consecutive repetitions of the back squat, followed by a 20 s transition interval and then eight consecutive maximal CMJ repetitions. All repetitions within each exercise were completed continuously without intra-set rest. A standardized inter-set rest period of 4 min was provided between complex sets, matching the recovery duration used in the cluster-set condition.

To minimize the influence of confounding factors, participants were instructed to maintain their habitual sport-specific wrestling training throughout the intervention period while refraining from any additional lower-body resistance or plyometric training outside the prescribed protocol. Training attendance was recorded for each session, and only participants who completed all scheduled training sessions were included in the final analysis.

### Statistical analysis

*A priori* power analysis was conducted using G*Power (v3.1.9.7) for a mixed-design repeated-measures ANOVA (group × time interaction). The effect size was estimated based on a previous study reporting partial eta squared (*ηp*^2^ = 0.21), which was converted to Cohen’s f (*f* = 0.52) (Rong & Xiu, 2024). With *α* = 0.05 and power = 0.95, the required total sample size was 16 participants (eight per group).

Continuous data were summarized using the mean ± standard deviation. The distributional characteristics of all variables were first examined with the Shapiro–Wilk test, and the outcomes of this assessment guided the choice of subsequent statistical analyses. Variables that satisfied the normality assumption were analyzed using parametric tests, whereas variables that violated normality in at least one group or time point were analyzed using non-parametric tests. Specifically, peak power, the 30-s squat kick test, and the 30-s hook-and-lift technique test required non-parametric analysis for at least one comparison; the remaining variables were analyzed using parametric methods. When variables conformed to the assumption of normality, paired-samples t-tests were conducted to assess within-group changes, while independent-samples t-tests were applied to compare differences between groups at both baseline and post-intervention time points. In contrast, variables that violated normality assumptions were analyzed using non-parametric methods, specifically the Wilcoxon signed-rank test for within-group comparisons and the Mann–Whitney U test for between-group comparisons. The threshold for statistical significance was defined as *p* < 0.05.

To further evaluate the overall effects of the intervention, including temporal changes between groups, a Generalized Estimating Equation (GEE) approach was adopted to analyze group × time interaction effects. This method is well suited for repeated-measures data and does not require strict distributional assumptions. Effect sizes were reported as the absolute values of Cohen’s d to aid interpretation of the practical magnitude of group × time effects (Tang et al., 2026). All statistical analyses were performed using IBM SPSS Statistics version 27 (IBM Corp., Armonk, NY, USA).

## Results

### Characteristics of the participants

At baseline, the participants’ mean age was 20.0 ± 2.1 years, and there were no significant differences in height, weight, and training experience (all *p* > 0.05). These results indicate that the two groups were well-matched in terms of demographic and training-related characteristics before the intervention (Table 1).

**Table 1:** Participant characteristics. Values are presented as mean ± standard deviation. Group comparisons at baseline were performed using independent-samples *t*-tests. No statistically significant differences were observed between groups for any baseline variable (*p* > 0.05).

**Parameter**	**CS-CT group (*n* = 9)**	**TS-CT group (*n* = 7)**	**Group** ***p***
Age (yrs)	20.44 (1.88)	19.57 (2.30)	0.417
Height (cm)	172.22 (6.44)	177.64 (5.51)	0.098
Weight (kg)	73.43 (12.36)	81.57 (15.92)	0.101
Training experience/year	6.56 (3.04)	6.29 (2.70)	0.592

**Notes.**

Values are presented as mean ± SD.

### Explosive performance

**Figure 3:**
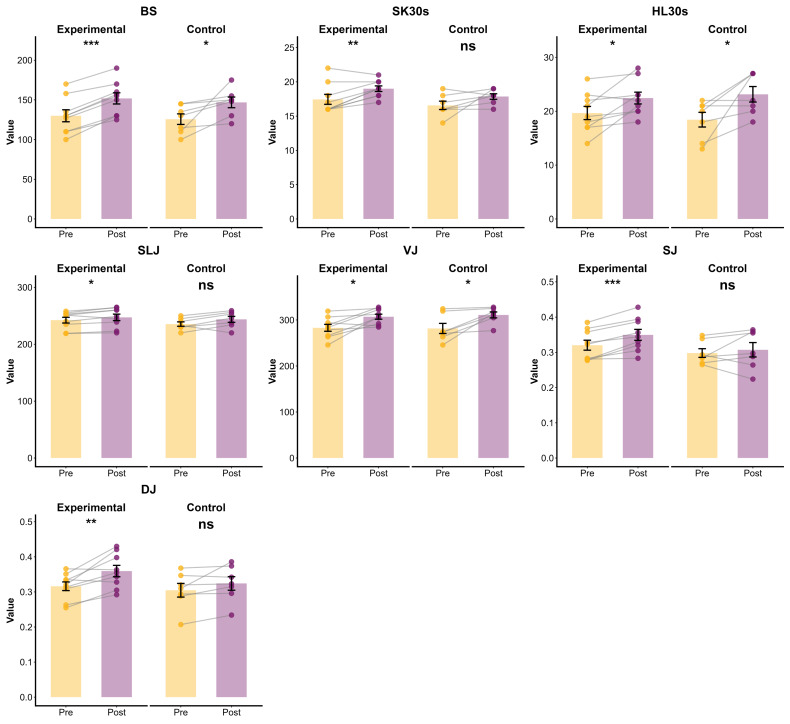
Pre–post changes in maximal strength, explosive performance, and sport-specific outcomes following 8-week complex training with different set configurations. Pre–post changes in maximal strength, explosive performance, and sport-specific performance following 8-week complex training with different set configurations. Bars represent group means, and error bars indicate standard error of the mean. Individual data points are shown, with lines connecting pre- and post-intervention values for each participant. BS, back squat 1RM; SK30s, 30-s squat kick test; HL30s, 30-s hook-and-lift test; SLJ, standing long jump; VJ, countermovement jump; SJ, squat jump; DJ, drop jump. Within-group changes are indicated as follows: *p < 0.05, **p < 0.01, ***p < 0.001; ns = not significant. No significant group × time interaction effects were observed for any variable (all *p* > 0.05).

Explosive power–related indicators showed varying degrees of change in both groups following the intervention (see Table 2 and Fig. 3). The CS-CT group demonstrated significant within-group improvements across multiple jump tests, whereas the TS-CT group exhibited significant changes in only a subset of indicators. Nevertheless, the group × time interaction effects for all lower-limb explosive power variables did not reach statistical significance.

**Table 2:** Pre- and post-intervention changes in strength, explosive performance, anaerobic power, and sport-specific performance in the CS-CT and TS-CT groups. Values are presented as mean ± standard deviation. Within-group changes were analyzed using paired-samples *t*-tests or Wilcoxon signed-rank tests depending on data distribution. Between-group differences at each time point were assessed using independent-samples *t*-tests or Mann–Whitney U tests. Group × time interaction effects were analyzed using generalized estimating equations (GEE). β represents the estimated interaction coefficient and CI represents the 95% confidence interval.

**Outcome**	**CS-CT group** **(*n* = 9)**	**TS-CT group** **(*n* = 7)**	***p***	**Group × Time**		
				β **(95% CI)**	***p***	***ES*** ^a^
Back squat 1RM (kg)						
Pre	130.00 ± 22.69	125.71 ± 17.66	0.687 (*t* = 0.411)	−0.75 (−15.71, 14.22)	0.922	0.040
Post	151.89 ± 21.11	146.86 ± 17.72	0.511 (*t* = 0.676)			
*p*	<.001 (*t*= −11.412)	0.039 (*t*= −2.639)				
Standing long jump distance (cm)						
Pre	242.33 ± 15.07	235.43 ± 10.23	0.317 (*t* = 1.089)	3.40 (−4.00, 10.79)	0.368	0.258
Post	247.22 ± 17.06	243.71 ± 13.94	0.658 (*t* = 0.453)			
*p*	0.019 (*t*= −2.927)	0.066 (*t*= −2.239)				
Vertical jump height (cm)						
Pre	282.92 ± 22.62	281.61 ± 29.12	0.921 (*t* = 1.808)	5.22 (−16.86, 27.29)	0.643	0.203
Post	307.06 ± 16.43	310.97 ± 17.03	0.649 (*t*= −0.465)			
*p*	0.012 (*t*= −3.225)	0.021 (*t*= −3.096)				
Squat jump height (m)						
Pre	0.32 ± 0.42	0.30 ± 0.33	0.272 (*t* = 1.142)	−0.02 (−0.05, 0.01)	0.150	0.056
Post	0.35 ± 0.47	0.307 ± 0.05	0.113 (*t* = 1.655)			
*p*	<.001 (*t*= −5.815)	0.538 (*t*= −0.654)				
Countermovement jump (m)						
Pre	0.36 ± 0.04	0.31 ± 0.05	0.092 (*t* = 1.759)	−0.01 (−0.42, 0.04)	0.980	0.216
Post	0.37 ± 0.05	0.33 ± 0.04	0.078 (*t* = 1.912)			
*p*	0.297 (*t*= −1.116)	0.478 (*t*= −0.756)				
Drop jump height (m)						
Pre	0.31 ± 0.04	0.30 ± 0.05	0.615 (*t* = 0.492)	−0.02 (−0.05, 0.01)	0.107	0.655
Post	0.36 ± 0.05	0.32 ± 0.05	0.181 (*t* = 1.398)			
*p*	0.006 (*t*= −3.664)	0.115 (*t*= −1.843)				
Mean power (W kg^−1^)						
Pre	7.53 ± 0.70	6.94 ± 1.01	0.190 (*t* = 1.315)	0.15 (−0.57, 0.87)	0.688	0.179
Post	7.5 ± 0.57	7.06 ± 0.35	0.088 (*t* = 1.942)			
*p*	0.884 (*t* = 1.51)	0.739 (*t*= −0.349)				
Peak power (W kg^−^^1^)						
Pre	11.69 ± 3.77	9.743 ± 1.45	0.289 (*Z* = − 1.060)	1.32 (−1.18, 3.82)	0.301	0.438
Post	10.40 ± 0.98	9.77 ± 0.51	0.121 (*t* = 1.664)			
*p*	0.477 (*Z* = − 0.711)	0.956 (*t*= −0.057)				
Fatigue index (%)						
Pre	61.46 ± 14.87	57.50 ± 4.51	0.468 (*t* = 0.754)	1.13 (−8.70, 10.96)	0.821	0.103
Post	61.02 ± 11.16	58.20 ± 3.68	0.494 (*t* = 0.710)			
*p*	0.929 (*t* = 0.091)	0.786 (*t*= −0.284)				
30-s squat kick test (reps)						
Pre	17.44 ± 2.19	16.57 ± 1.62	0.571 (*Z* = − 0.566)	−0.27 (−1.80, 1.27)	0.730	0.135
Post	19.00 ± 1.23	17.86 ± 1.07	0.067 (*t* = 1.990)			
*p*	0.019 (*Z* = − 2.345)	0.122 (*t*= −1.80)				
30-s hip-rotation kick test (reps)						
Pre	19.67 ± 3.67	18.43 ± 3.60	0.510 (*t* = 0.676)	1.94 (−1.92, 5.80)	0.325	0.527
Post	22.44 ± 3.30	23.14 ± 3.80	0.706 (*t*= −0.394)			
*p*	0.025 (*t*= −2.748)	0.44 (*t*= −2.533)				
30-s hook-and-lift technique test (reps)						
Pre	32.67 ± 19.50	31.00 ± 0.378	0.703 (*Z* = − 0.381)	−1.33 (−4.42, 1.75)	0.397	0.086
Post	35.00 ± 2.26	32.00 ± 1.09	0.287 (*Z* = − 1.066)			
*p*	0.080 (*Z* = − 1.749)	0.518 (*Z* = − 0.647)				

**Notes.**

Data are presented as mean ± SD. t indicates parametric tests, and Z indicates non-parametric tests.

a Effect sizes were reported as the absolute values of Cohen’s d.

Standing long jump performance improved significantly from baseline in the CS-CT group (*p* = 0.019), while no significant change was observed in the TS-CT group (*p* = 0.066), with a non-significant group × time interaction effect (β = 3.40, 95% CI [4.00–10.79], *p* = 0.368). Countermovement jump height increased significantly in both the CS-CT group (*p* = 0.012) and the TS-CT group (*p* = 0.021), whereas squat jump height showed a significant improvement only in the CS-CT group (*p* < 0.001), with no significant change in the TS-CT group (*p* = 0.538). The group × time interaction effects for CMJ (β = 5.22, 95% CI [16.86–27.29], *p* = 0.643) and SJ (β = −0.02, 95% CI [0.05–0.01], *p* = 0.150) were not significant. In addition, drop jump height increased significantly from baseline in the CS-CT group (*p* = 0.006), while no significant change was observed in the TS-CT group (*p* = 0.115), and the corresponding group × time interaction effect did not reach statistical significance (β = −0.02, 95% CI [0.05–0.01], *p* = 0.107). Effect sizes for group × time comparisons in explosive performance outcomes were small to moderate, ranging from 0.056 to 0.655, with the largest effect observed for drop jump height.

### Anaerobic power

Neither the CS-CT group nor the TS-CT group exhibited significant within-group differences in anaerobic power outcomes, and no significant group × time interaction effects were observed across all Wingate-derived variables (see Table 2 and Fig. 3).

Specifically, mean power, peak power, and fatigue index did not change significantly from baseline in either the CS-CT group or the TS-CT group (all *p* > 0.05). Consistently, the group × time interaction effects for mean power (β = 0.15, 95% CI [0.57–0.87], *p* = 0.688), peak power (β = 1.32, 95% CI [1.18–3.82], *p* = 0.301), and fatigue index (β = 1.13, 95% CI [8.70–10.96], *p* = 0.821) did not reach statistical significance. Effect sizes for Wingate-derived outcomes were small to moderate, ranging from 0.103 to 0.438.

### Sport-specific performance

Sport-specific performance indicators exhibited selective within-group improvements following the intervention (see Table 2 and Fig. 3). The CS-CT group demonstrated significant improvements in some wrestling-specific tests, whereas the TS-CT group showed significant changes in fewer indicators. However, no significant group × time interaction effects were observed across all sport-specific performance measures.

Specifically, performance in the 30-s squat kick test improved significantly from baseline in the CS-CT group (*p* = 0.019), while no significant change was observed in the TS-CT group (*p* = 0.122), with a non-significant group × time interaction effect (β = −0.27, 95% CI [1.80–1.27], *p* = 0.730). The 30-s hip-rotation kick test showed significant within-group improvements in both the CS-CT group (*p* = 0.025) and the TS-CT group (*p* = 0.044); however, the corresponding group × time interaction effect was not statistically significant (β = 1.94, 95% CI [1.92–5.80], *p* = 0.325). In contrast, no significant pre–post changes were observed in the 30-s hook-and-lift technique test in either group (CS-CT: *p* = 0.080; TS-CT: *p* = 0.518), and the group × time interaction effect was not significant (β = −1.33, 95% CI [4.42–1.75], *p* = 0.397). Effect sizes for sport-specific performance outcomes were small to moderate, ranging from 0.086 to 0.527, with the largest effect observed for the 30-s hip-rotation kick test.

### Maximal strength

Maximal strength showed significant improvements in both groups following the intervention (see Table 2 and Fig. 3). Both the CS-CT and TS-CT groups demonstrated marked increases in back squat one-repetition maximum; however, no significant group × time interaction effect was observed.

Specifically, back squat 1RM increased significantly from baseline in the CS-CT group (*p* < 0.001) and in the TS-CT group (*p* = 0.039). Consistently, the group × time interaction effect for back squat 1RM did not reach statistical significance (β = −0.75, 95% CI [15.71–14.22], *p* = 0.922). The corresponding effect size was small (ES = 0.040).

## Discussion

The primary findings indicated that, after an 8-week intervention, both CS-CT and TS-CT induced significant improvements in maximal lower-limb strength, explosive performance, and sport-specific outcomes. These results demonstrate that CT is an effective training modality for enhancing physical performance in highly trained wrestlers. However, no significant group × time interaction effects were observed across any primary outcome, indicating that, under the present conditions, differences in set configuration did not translate into clearly distinguishable long-term training adaptations. but, the possibility of Type II error cannot be excluded, particularly if the true differences between set configurations are small to moderate. Therefore, the absence of statistically significant differences should not be interpreted as definitive evidence of equivalence between the two training models.

From an applied perspective, these findings first highlight that, when training load is rigorously controlled, CT itself represents a stable and effective conditioning strategy for elite wrestlers (Cormier et al., 2020; Marshall et al., 2021). Regardless of whether CS-CT or TS-CT was implemented, athletes exhibited practically meaningful improvements in lower-limb maximal strength (back squat 1RM), jump-based explosive performance (SJ, DJ, and SLJ), and sport-specific tests (*e.g.*, 30-s squat kick and 30-s hook-and-lift drills). This suggests that, in national-level athletes, when the overall training stimulus remains within an effective range, the integrated mechanical and neuromuscular demands imposed by CT are sufficient to drive concurrent improvements across multiple performance domains (Blazevich & Babault, 2019; McKay et al., 2022).

Beyond these general effects, the present study further indicates that, within the applied training program and participant characteristics examined, modifying set configuration alone within a CT framework was insufficient to alter long-term physical adaptations (Cormier et al., 2020; Rong & Xiu, 2024). Specifically, the CS-CT protocol employed in this study involved subdividing a traditional continuous set into multiple smaller training units, rather than introducing classical intra-set rest intervals between consecutive repetitions of the same exercise (Jukic et al., 2022; Tufano, Brown & Haff, 2017). Therefore, the present findings should be interpreted as applying to this redistributed complex-training configuration rather than to all classical cluster-set models. Although this “distributed” organization altered the pacing within a session, it may not have produced a fundamentally different accumulation of fatigue during the squat exercise compared with TS-CT (Bakhshinejad et al., 2025; Jukic et al., 2020). As a result, the average execution quality of explosive actions, such as vertical jumps, may have remained comparable between groups across the training period (Jukic et al., 2020; Tufano et al., 2016).

In addition, the participants were national-level wrestlers with extensive systematic training backgrounds, characterized by well-developed strength capacities and high fatigue tolerance (Chaabene et al., 2017; McKay et al., 2022). In such training-mature athletes, when total volume and absolute intensity are tightly matched, the stimulus differences introduced by set configuration—a relatively fine-grained programming variable—may be insufficient to exceed existing adaptive thresholds (Bishop, Jones & Woods, 2008; Cormie, McGuigan & Newton, 2011; Issurin, 2010). Consequently, these differences are unlikely to manifest as statistically distinguishable adaptations over an 8-week time frame (Mujika et al., 2018; Rhea et al., 2003). It is also important to consider that the intervention was conducted during the competitive phase, where fatigue management and performance maintenance are typically prioritized over large performance gains. In this context, although both set configurations produced comparable improvements, the cluster-set approach may offer practical advantages by potentially reducing perceived fatigue and preserving movement quality, making it a potentially more suitable strategy during competition periods. In addition, the relatively small sample size (*n* = 16), although typical for studies involving national-level athletes, should be considered when interpreting the absence of significant group × time interaction effects. However, this athlete-specific context may also limit the generalizability of the findings to less-trained populations, in whom adaptive margins and responsiveness to programming variables may differ. While the *a priori* power analysis indicated that this sample size was sufficient to detect moderate-to-large effects, the study may have been underpowered to identify smaller, yet practically meaningful, interaction effects between set configurations over time. This limitation is particularly relevant in highly trained populations, where inter-individual variability is often reduced and adaptive changes tend to be more subtle. Therefore, the lack of significant interaction effects should be interpreted with caution. This observation suggests that, in elite populations, modest structural modifications within a training program may not substantially alter the overall trajectory of physical development during a medium-term training cycle (Bourdon et al., 2017; Kiely, 2012).

It should be acknowledged that movement velocity, power output, and acute fatigue during squat execution were not directly monitored in this study (Weakley et al., 2021). Therefore, it cannot be determined whether different set configurations produced sustained differences in execution quality during training (Latella et al., 2019). Previous studies have suggested that CS may enhance training outcomes by preserving movement velocity and power output; however, if such advantages were not consistently expressed during actual training sessions, the convergence of long-term outcomes observed here is theoretically plausible (Jukic et al., 2020; Tufano et al., 2016). Accordingly, the present findings primarily reflect integrated long-term adaptations rather than acute, session-level neuromuscular responses (Cormier et al., 2022; Garbisu-Hualde & Santos-Concejero, 2021). This interpretation is consistent with previous cluster-set research showing that the main advantages of cluster configurations are often observed at the acute session level, including reduced mechanical fatigue, better maintenance of movement velocity, and lower perceptual or metabolic stress (Jukic et al., 2022; Latella et al., 2019; Tufano et al., 2016). In the present study, however, internal-load and neuromuscular variables during training were not directly monitored. Therefore, although both groups completed the same external training load, it remains unclear whether the CS-CT protocol actually produced lower internal load or superior neuromuscular quality during training.

When contextualized within the existing literature, the present results—demonstrating that CT effectively enhances strength, explosive performance, and sport-specific capacity—are consistent with previous systematic reviews and meta-analyses (Bauer et al., 2019; Freitas et al., 2017; Thapa et al., 2021; Wang et al., 2023). Prior evidence generally supports the efficacy of CT across a range of sports (Freitas et al., 2017; Thapa et al., 2021). In contrast, reported advantages of CS have predominantly been derived from acute or short-term studies focusing on movement velocity, power output, or fatigue-related responses (Latella et al., 2019). Investigations embedding CS within CT frameworks and examining long-term performance outcomes remain limited (Rong & Xiu, 2024). By directly comparing CS-CT and TS-CT under rigorously matched training loads in an applied setting, the present study provides context-specific evidence regarding the practical relevance of set configuration in elite athletes.

In summary, under conditions of strictly controlled training volume and progressive intensity, both CS-CT and TS-CT over 8 weeks elicited effective and comparable improvements in maximal lower-limb strength, explosive performance, and sport-specific capacity in national-level male Chinese-style wrestlers (Cormier et al., 2020; Rong & Xiu, 2024). These findings suggest that, in elite wrestlers, the overall loading strategy of CT may be more influential than subtle differences in set configuration (Kiely, 2012). Future studies should incorporate direct monitoring of movement velocity, power output, and fatigue-related markers during training, and extend intervention durations or examine different training phases, to clarify the conditions under which set-configuration manipulations may exert more differentiated effects on long-term adaptations (Cormier et al., 2022; Mujika et al., 2018; Weakley et al., 2021).

## Limitations

Two limitations of the present study should be acknowledged when interpreting the findings. First, although the intervention was conducted under rigorously controlled conditions with matched training volume and progressive intensity, the relatively small sample size—inevitable when working with national-level athletes—may have limited the statistical power to detect subtle group × time interaction effects (Atkinson & Batterham, 2015; McKay et al., 2022). This constraint is common in elite sport research; nevertheless, future studies employing multi-center collaboration may help increase sample size and enhance statistical sensitivity (Jukic et al., 2020; Weakley et al., 2021).

Second, acute neuromuscular and mechanical variables during training sessions—such as barbell velocity, power output, velocity loss, and perceptual fatigue—were not directly monitored. As a result, it remains unclear whether the two set configurations induced different execution qualities or fatigue profiles at the session level, which may have contributed to the absence of clearly differentiated long-term adaptations between groups (Jukic et al., 2020; Latella et al., 2019). Future investigations incorporating real-time velocity-based or wearable monitoring technologies may provide clearer insight into the relationship between acute training responses and chronic performance adaptations (Bourdon et al., 2017; Cormier et al., 2022).

Third, although external training-load variables were deliberately matched between groups to reduce confounding, this control may also have limited the physiological distinction between the two protocols. In addition, the CS-CT protocol used in this study should be interpreted as a redistributed complex-training configuration rather than a classical intra-set cluster model. Because internal-load and acute neuromuscular variables were not directly monitored, it remains unclear whether the intended differences between protocols were fully achieved during training. Finally, the 20-s transition between the squat and CMJ may have limited optimal PAPE expression, as residual fatigue may have attenuated or masked potential potentiation effects.

Finally, although the Wingate test is a widely used measure of anaerobic performance, it may not fully capture the intermittent, multidirectional, and technically constrained anaerobic demands of wrestling. This may partly explain the limited changes observed in Wingate-derived variables and should be considered when interpreting the anaerobic performance results.

## Conclusion

The results of this randomized, volume-matched trial indicate that, within a tightly controlled training framework—where total training volume, exercise selection, and progressive intensity were strictly matched—the overall training stimulus provided by complex training appears to be the primary driver of physical adaptations, rather than the finer structural variable of set configuration (Cormier et al., 2020; Kiely, 2012). However, because internal-load responses were not monitored and the physiological differentiation between protocols may have been limited, these findings should be interpreted cautiously rather than as definitive evidence that set configuration has no effect. In highly trained athletes with well-developed strength and fatigue tolerance, the differences in intra-set recovery associated with set-configuration manipulation may not result in clearly differentiated long-term performance enhancements over a medium-term training cycle (Issurin, 2010).

Consequently, from a practical perspective, strength and conditioning practitioners working with elite wrestlers may prioritize the implementation of well-structured, progressively loaded complex training programs, without necessarily considering set configuration as a decisive factor for enhancing long-term adaptations (Bourdon et al., 2017; Cormier et al., 2022).

##  Supplemental Information

10.7717/peerj.21438/supp-1 Supplemental Information 1 Raw data for complex training study Raw data used for statistical analysis in the study examining the effects of cluster-set and traditional-set complex training in national-level wrestlers.

## Abbreviations

1RM: One-Repetition Maximum
ANOVA: Analysis of Variance
AP: Average Power
CI: Confidence Interval
CMJ: Countermovement Jump
CS: Cluster Set
CS-CT: Cluster-Set Complex Training
CT: Complex Training
DJ: Drop Jump
GEE: Generalized Estimating Equation
PAP: Post-Activation Potentiation
PAPE: Post-Activation Performance Enhancement
PD: Power Drop (Fatigue Index)
PP: Peak Power
SJ: Squat Jump
SLJ: Standing Long Jump
TS: Traditional Set
TS-CT: Traditional-Set Complex Training

## Additional Information and Declarations

### Data Availability

The following information was supplied regarding data availability: The raw measurements are available in the Supplemental File.

### Clinical Trial Registration

The following information was supplied regarding Clinical Trial registration: 2025159
